# Comparative analysis of sandy beach and foredune geomorphic change measurements from Apple lidar and small-unoccupied aerial systems

**DOI:** 10.1038/s41598-024-63466-1

**Published:** 2024-06-04

**Authors:** Brendan M. J. Burchi, Ethan J. Theuerkauf

**Affiliations:** Department of Geography, Environment, and Spatial Sciences, Michigan State University, 673 Auditorium Road, East Lansing, MI 48824 USA

**Keywords:** Geomorphology, Natural hazards

## Abstract

Sandy beach and foredune environments are common throughout coastlines globally. Coastal landscapes are dynamic and vulnerable to water level fluctuations, storm events, and human disturbances. Standard methods for measuring geomorphic changes include small-unoccupied aircraft systems paired with structure-from-motion photogrammetry (sUAS-SfM), but this can be costly and logistically challenging. We evaluated the accuracy of Apple lidar in comparison to high precision sUAS-SfM and RTK-GPS to map sandy beach and foredune geomorphic change. Checkpoint elevations were measured via RTK-GPS and both the sUAS-SfM and Apple lidar surveyed elevations were compared against these checkpoints to evaluate the performance of both methods for measuring elevations. The sUAS-SfM elevation data were on average around 0.004 m above/below the checkpoint elevations while the Apple Lidar elevations were around 0.039 m. Apple lidar and sUAS-SfM-derived volumetric measurements and spatial patterns of erosion and accretion were compared to evaluate the Apple lidar’s ability to detect geomorphic change over time. The geomorphic changes documented from these two methods were similar though the Apple lidar appeared to capture finer-scale erosion and accretion patterns. Our findings indicate that the Apple lidar can capture sandy beach and foredune geomorphic changes rapidly and accurately, which can promote proactive and resilient coastal management.

## Introduction

Sandy beaches and foredunes are found throughout the world and are critically important systems from ecological and economic perspectives^1^. About one third of the world’s ice-free shorelines are comprised of sandy beaches and foredunes^2^. These landforms are a management priority for local, state, and federal decisionmakers. Proactive and successful management requires high-quality and timely data on coastal change^3–5^ which can be challenging to acquire given the dynamic nature of beach and foredune systems. Numerous methods exist for mapping coastal change, ranging from relatively simple and low-cost GPS surveys to high spatial resolution and costly lidar scanners^3,6–9^. Small-unoccupied aerial systems (sUAS), which can range from low-cost systems (hundreds of USD) to expensive platforms (thousands of USD), and structure from motion (SfM) photogrammetry have recently been at the forefront of coastal mapping, however, logistical constraints such as licensing, permitting, and weather can restrict sUAS-SfM data collection^3,7–9^. To date, these methods are generally only accessible to scientists and survey professionals yet new technologies, such as the Apple lidar, are expanding access to these technologies^10^. While accessible, few studies have quantitatively evaluated the performance of the Apple lidar for mapping geomorphic change. Here, we present an accuracy assessment of the Apple lidar for monitoring geomorphic change at sandy beach and foredune systems.

An exposed sandy beach and foredune system is subject to a wide range of geomorphic forces that induce change across a range of spatiotemporal scales. Water level changes, such as rising sea level or fluctuating lake levels, inundate these coastal landscapes and shift the zone of wave influence landward or basinward^11,12^. Waves are the primary agent of change for beaches and dunes and are principally responsible for inducing erosion and offshore transport of sand from these systems^13–15^. In addition to erosion, these waves and associated currents, such as longshore currents, move sediment throughout the nearshore^16,17^. Low-energy wave conditions can result in onshore transport of material, potentially leading to beach accretion^18,19^. In some coastal areas, where wide beaches with fine-grained material occur, aeolian (wind-blown) sand transport can be an important driver of geomorphic change^20,21^. Onshore winds lead to aeolian transport of material from the beach, resulting in the formation and evolution of foredunes^22^. These foredunes can continue to grow provided there is sufficient sand supply for aeolian transport^23^. If water levels increase or large storm events occur, these foredune systems are highly susceptible to erosion and will transport material either back to the beach or out into the nearshore^24^. Over time, coastlines will erode or accrete in response to the cumulative effect of these processes, which necessitates management decisions to preserve landscape form and/or function.

Mapping and monitoring these changes are imperative for coastal processes research as well as management decision-making. The dynamic nature of coastal morphology requires that monitoring approaches be rapidly deployed before and after storm events^25^ or other coastal hydrodynamic events such as seiches^26^ or sea-level anomalies^27^. Furthermore, increased anthropogenic pressure on coastal areas necessitates efficient mapping approaches both for monitoring the impacts after a management action (e.g., dune restoration or beach nourishment) as well as to document vulnerable locations where engineering or other management solutions are needed. Given the wide range of temporal and spatial scales that monitoring must encompass, a variety of monitoring approaches are now available for documenting coastal change.

Modern methods to map and monitor coastal morphology include high accuracy real-time kinematic (RTK) GPS, terrestrial and airborne lidar, and sUAS-SfM^3,28^. Differences in these methods include cost, feasibility, and spatial and temporal resolution^29–31^. The Apple lidar offers a solution that strikes a balance between cost, accessibility, and high spatiotemporal resolution. Several studies have shown the Apple lidar applied within disciplines ranging from anthropology, terrestrial ecology, and cryosphere science. Within these domains the lidar scanner has been used to document cultural artifacts in 3D^32,33^, inventory trees in forests^34^, and monitor snow depth changes^35^.

Despite the variety of documented applications for the Apple lidar in other scientific fields, this technology has only been applied in the coastal realm to monitor cliff changes^10^. This study tested the accuracy and precision of the Apple iPhone 12 Pro and iPad Pro lidar by finding similarities between the dimensions of scanned meshes and real values of rectangular boxes before measuring a coastal cliff in eastern Denmark. The study determined that although the accuracy and precision of the Apple lidar does not reach high-resolution point cloud structure-from-motion standards, it is possible to represent coastal cliff environments to within ± 10 cm. Additionally, they determined that the scanners on both the iPhone and iPad are identical in terms of resolution, and the device’s orientation and angle have no impact on the resulting topographic measurements.

Our study evaluated the application of the Apple lidar for monitoring sandy beach and foredune geomorphic changes. First, topographic maps generated at a sandy beach and foredune site using the Apple lidar were compared to sUAS-SfM-derived maps. The aim of this comparison was to determine whether the Apple lidar can produce topographic maps that are as accurate as the established sUAS-SfM technology. Then, temporal changes in beach and foredune morphology were evaluated using both the Apple lidar and sUAS-SfM to explore whether this new technology can be used to document geomorphic changes. The results from this study were then placed in context with their implications for both scientific research as well as coastal management and decision-making.

## Methods

**Figure 1:**
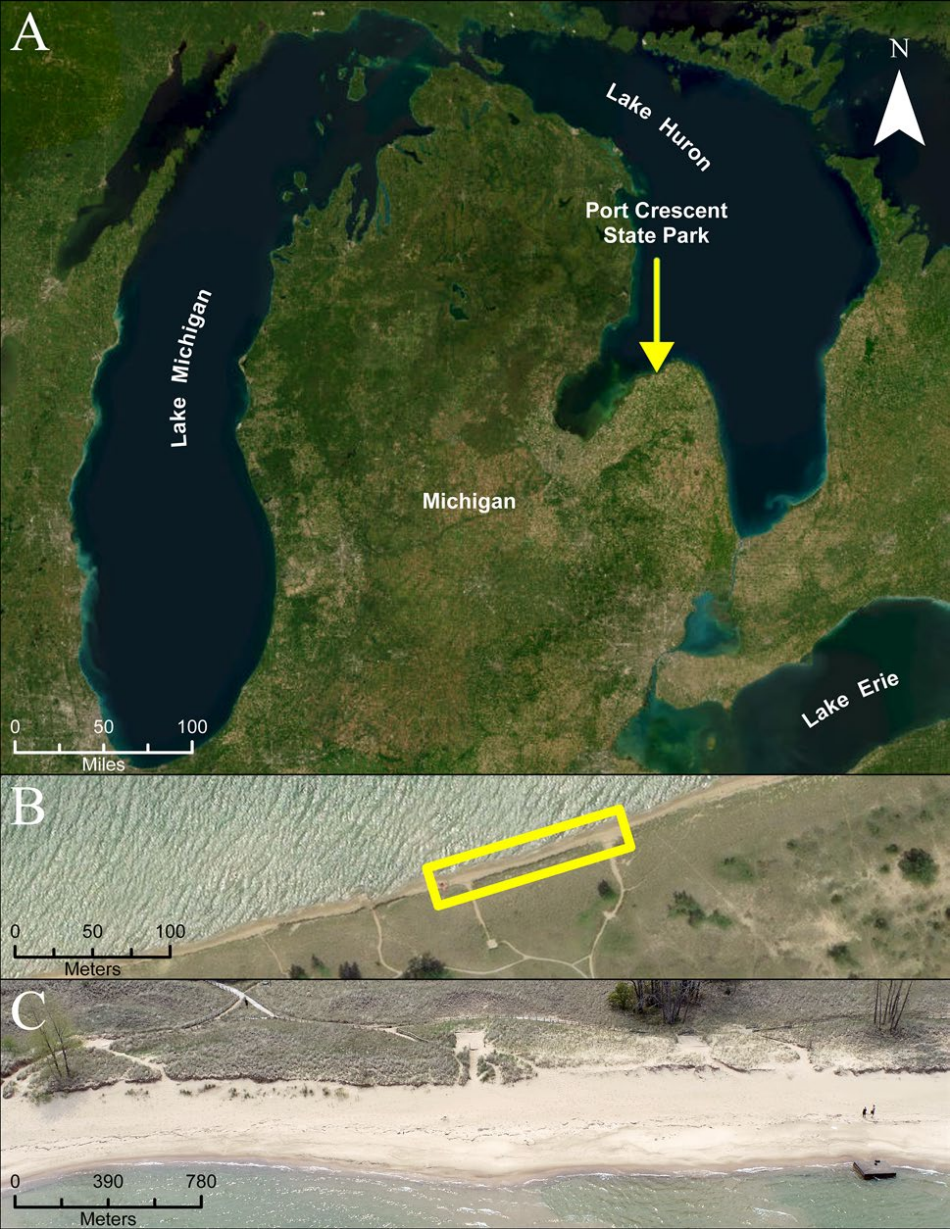
(**A**) Location of PCSP along the western shore of Lake Huron; (**B**) spatial extent of focus site within PCSP; (**C**) oblique image of the focus site collected on 07/18/2023 depicting the wide sandy beach and foredune environment.

Data were collected for this study on July 18, 2023, and September 01, 2023, when wind was minimal (less than 7 mph) at Port Crescent State Park (PCSP), which is located on Lake Huron in Michigan (Fig. 1). PCSP is a sandy beach and foredune site located in the middle of an embayment and is free of any shore protection structures that may alter natural coastal processes. Given this, it is an ideal location to test the utility of the Apple lidar for mapping and monitoring beach and foredune geomorphic change.

### sUAS-SfM data collection

**Figure 2:**
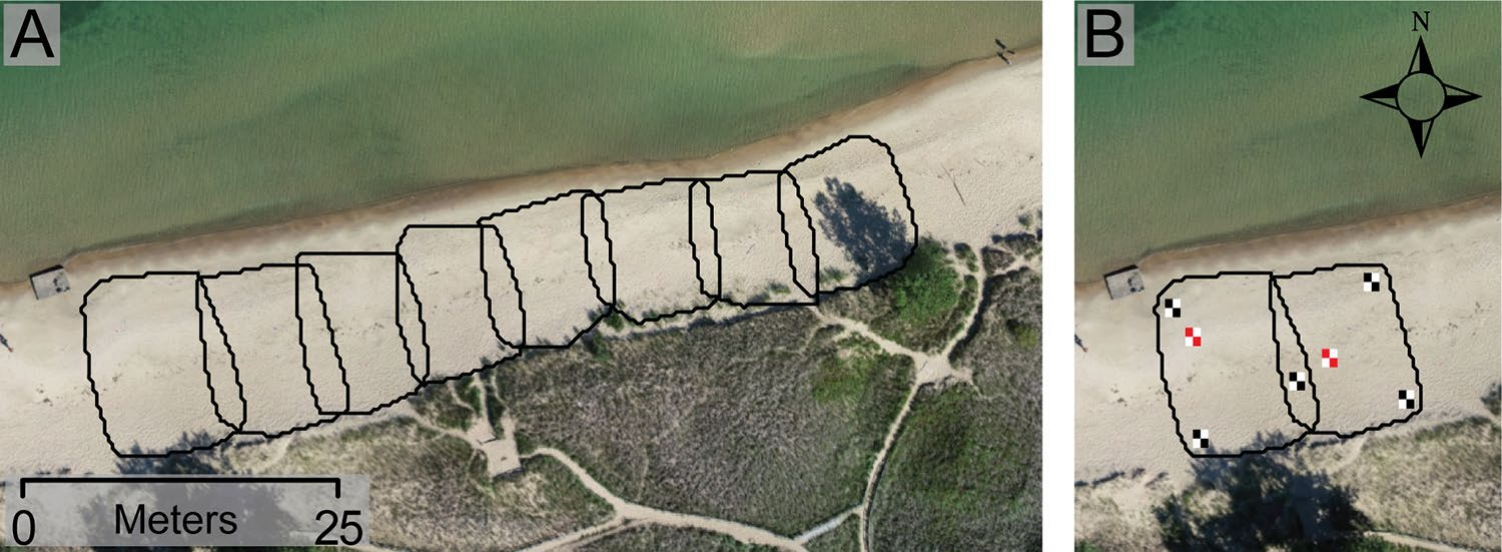
(**A**) Spatial footprint of the Apple lidar scans from the September 2023 survey detailed the extent and overlap of the scans; (**B**) Example of GCP and CP placement for two successive scans; note that each scan has 3 GCPs and 1 CP and that one of the GCPs is common between both scans.

Aerial photographs were acquired from a DJI Phantom 4 Pro V2.0 equipped with a gimbal-mounted camera setup. Controlled by the DJI Go 4 and the DJI Ground Station Pro applications, the sUAS-SfM flew a predetermined mission and captured high-resolution aerial imagery with 80% front and side overlap. Only nadir images were collected for analysis and the sUAS-SfM flew at an altitude of ~ 55 m, which yielded a ground sampling distance of 1.5 cm/pixel. Ground control points (GCPs) and checkpoints (CPs) were placed throughout the site and surveyed with a Trimble R10-2 RTK-GPS (~ 2 cm horizontal and vertical accuracy, Supplementary Fig. S1, S2). Both GCPs and CPs were distributed throughout the site to capture the variations in elevation and were used to spatially reference the derived spatial data (GCPs) and to determine horizontal and vertical errors of the data products (CPs). In the July 2023 survey 11 GCPs and 8 CPs were placed and in the September 2023 survey 13 GCPs and 8 CPs were placed. The GCPs used were a 0.3 m × 0.3 m black and white tile and the CPs were a 0.3 m × 0.3 m red and white tile (Fig. 2). In total, the time to place GCPs, CPs, and complete the sUAS flight took roughly forty-five minutes.

### Apple lidar data collection

A similar precision test to the coastal cliff study was conducted with the Apple lidar by comparing scanned measurements with physical measurements (with a tape measure) of a rectangular box. The dimensions of the box measured 45.5 × 41 × 31.5 cm (L × W × H) with a tape measure. The box dimensions were measured from the lidar scan using the 3D Scanner application version 2.3.1. With the measure tool in the app, four individuals took measurements of the length, width, and height by placing points along the outline of the scanned box. (Supplementary Table S1). These measurements were all within 90% percent of the physical measurements.

Apple lidar scans were completed at the focus site with an Apple iPad Pro 4th generation using the 3D Scanner app (Fig. 2). Within the application, the “Lidar Advanced” setting was selected rather than the default setting to allow for advanced controls described below, but the quality of data remained the same. The advanced controls for the survey were as follows: a max laser range of 5.0 m, spatial resolution of 10 mm to enhance detail, high confidence, and no masking. The high confidence setting records data by quality, filtering out and saving only the highest quality data returns and the masking setting defines limits on what the scanner will measure during data collection. If no masking is selected, the 3D scanner application records everything visible from the lidar scanner^30^.

In the field, scans were collected by pacing perpendicular to the shoreline and foredune at 1–2 m/s. While the direction of scanning the landscapes morphology does not matter, the speed does. Moving too fast can result in lower data quality for the 3D Scanner app rendered point cloud. One scan was not possible to cover the area of the desired focus site because of storage constraints in the 3D Scanner app, so a series of eight scans were collected that were all roughly 400 m^2^. Within the scan boundaries (Fig. 2) a minimum of three GCPs were needed to ensure spatial accuracy and we ensured that there was at least one CP to validate the data. The GCPs were strategically placed to allow for correct spatial orientation and overlap between scans, and CPs were randomly set at varying elevations. The GCP overlap aids in mosaicking the scans together to create a single Apple lidar DEM. Each scan took five to ten minutes to collect and a further two minutes to process in high definition. The sUAS-SfM survey was conducted immediately prior to the Apple lidar survey on both July 18, 2023 and September 01, 2023. Less than one hour occurred between the survey therefore both methods captured the same beach morphology.

### Data processing

The imagery collected from the sUAS were imported into Agisoft Metashape for structure-from-motion (SfM) photogrammetric processing following established techniques outlined in^3^. Images were initially evaluated for image quality with poor quality images (quality value less than 0.5) being removed before alignment and further processing. Once the photos were aligned, the GCPs were placed in the images to spatially reference the imagery and the CPs were placed to evaluate the accuracy of the resulting elevation model. A dense point cloud was then built from the spatially referenced and aligned imagery. This dense point cloud is used to construct the orthomosaic images and the DEMs that were used as the comparison datasets to the Apple lidar data. The total checkpoint error for the July 2023 dataset was 0.042 m and the September 2023 dataset was 0.025 m. The DEMs were exported from Metashape as ASCII grids with a 0.5 m grid spacing.

A vertical change detection threshold (minimum level of vertical change that can be detected) was used for the spatial and temporal comparisons between survey methods. This threshold was calculated by combining the average vertical precision of the original GCP survey points from the RTK-GPS and the total sUAS-SfM GCP error from Agisoft Metashape. For July, the GCP vertical precision averaged to be 0.024 m and the GCP error was 0.014 m. For September, the vertical precision averaged to be 0.024 m and the GCP error was 0.02 m. This results in a total vertical change threshold for July of 0.038 m and 0.044 m for September. We used the higher vertical change detection threshold, and rounded up to the nearest centimeter to create an error threshold of ± 0.05 m.

**Figure 3:**
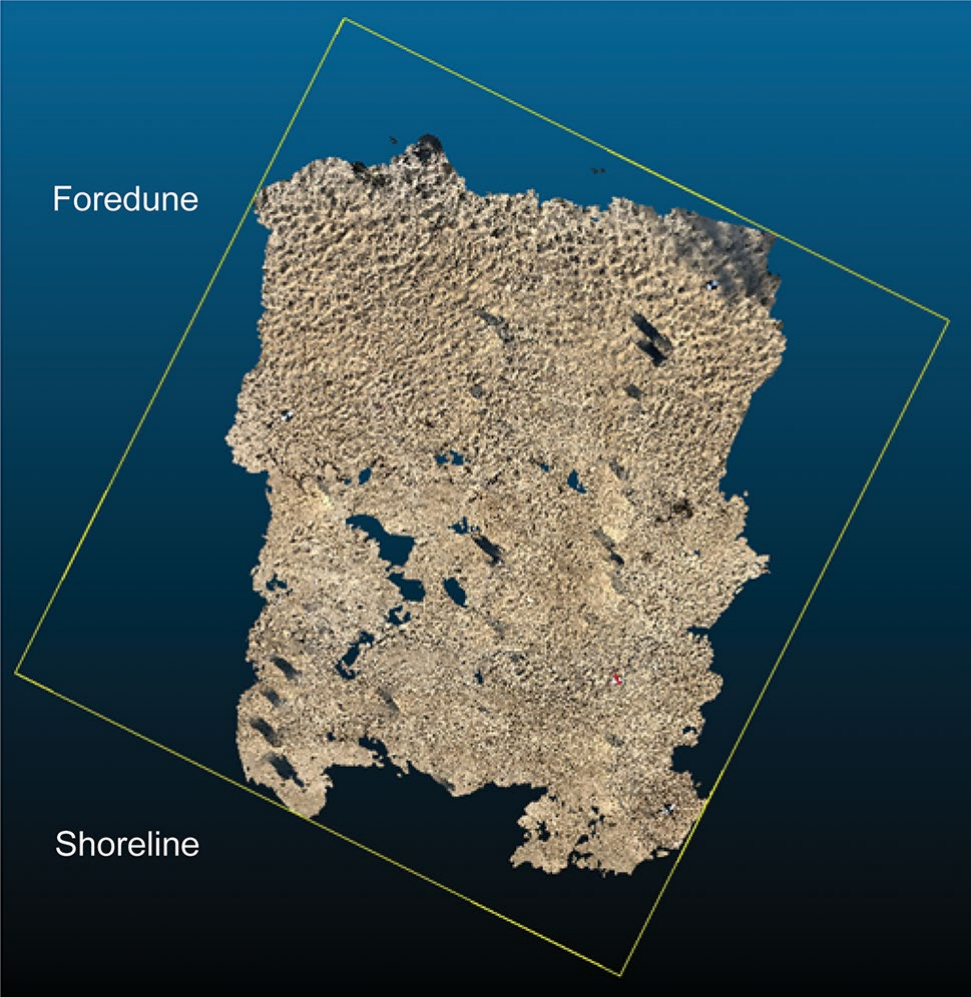
Ungeoreferenced LAS point cloud of Apple lidar scan 1 in m, 09/01/2023 in CloudCompare. The yellow bounding box line contains the point cloud for this scan which is comprised of 11,403,513 data points.

The scans collected with the Apple lidar were initially processed to improve detail, precision, and texture quality within the 3D Scanner application and then exported as laser aerial survey files (LAS). These LAS files were imported into CloudCompare, which is a 3D point cloud processing and analysis software package, for georeferencing and point cloud alignment (Fig. 3). The horizontal and vertical position information is input into the corresponding marker within the point cloud for the surveyed GCPs. Once the GCP information is entered, the point cloud is aligned. These steps were repeated for all eight of the scans that were collected on each survey date. All these georeferenced point clouds were exported out of CloudCompare as ASCII XYZ files for additional processing.

The ASCII XYZ files were uploaded into Golden Software’s Surfer program for gridding and analysis. The XYZ data were gridded using Kriging Interpolation and a grid spacing of 0.5 m was selected to match the spacing of the sUAS-SfM-derived DEMs. The resulting grids (8 per survey date) were then mosaicked together within Surfer to create one seamless DEM for each survey date. The spatial extents of the Apple lidar DEMs did not exactly match the sUAS-SfM DEMs therefore, the models were clipped to a common extent that only included the maximum overlap of the models for each survey date.

This sUAS-SfM-derived DEM was subtracted from the Apple lidar-derived DEM using Surfer’s Grid Math function (differencing the elevation points within each grid node) to create a DEM of Difference (DOD) that was used to evaluate the spatial pattern of elevation differences between the two DEMs. Profile data were extracted from both DEMs as well to explore any differences in elevation and morphology reconstruction between the Apple lidar and the sUAS-SfM in the cross-shore dimension. Additionally, the CP elevations collected with the RTK-GPS (Z) were compared to the elevations at those XY locations within the Apple lidar- and sUAS-SfM-derived DEMs (Z_1_) by subtracting Z from Z_1_. The DEMs experimental values (Z_1_) were extracted in Surfer from the XY location of the original RTK-GPS surveyed CP elevation (Z). The intent of this comparison was to confirm that the Apple lidar can generate accurate elevation data as compared to standard topographic mapping methods like RTK-GPS and sUAS-SfM surveys.

Once accuracy of the Apple lidar DEMs was documented, a comparison between two successive surveys using both the Apple lidar and the sUAS-SfM was conducted. DODs were created for each method between the July and September 2023 surveys. The aim of this analysis was to document the ability of the Apple lidar to map geomorphic change at a sandy beach and foredune system. The spatial patterns of mapped erosion and accretion were first qualitatively compared between both methods to document whether there were visual differences in the captured changes. Then, the volume changes between survey dates were derived for both methods and compared as a quantitative metric of differences between methods. There are differences in the net area for the calculations of the DOD volume measurements and therefore cannot be interpreted directly. A ratio was calculated that represents the volume of sediment moving within the system, calculated as sediment volume (m^3^) divided by total area (m^2^) measured.

## Results

### Comparison of elevation measurements to RTK-GPS

Elevation accuracies of the Apple lidar and sUAS-SfM models were compared by analyzing the derived elevation value (Z_1_) from both survey methods against the RTK-GPS surveyed elevation (Z) for sixteen total CPs (Table 1). The largest differences between the lidar measured elevation and the RTK-GPS were July CP 1, which was 0.11 m below the RTK-GPS surveyed elevation (Z), and September CP 5 0.11 m above. In contrast, the largest elevation differences measured for sUAS-SfM were September CP 2, which was 0.049 m below the RTK-GPS and July CP 7, which was 0.089 m above. The Apple lidar exhibited larger elevation differences than sUAS-SfM for five of the eight CPs in both data sets resulting in the average lidar elevation measurements being about five times higher than the sUAS-SfM average. Of the sixteen CPs, nine measured by sUAS-SfM and fourteen surveyed by Apple lidar fell below (sUAS-SfM average = −0.028 m, Apple lidar average = −0.052 m; Table 1) the original RTK-GPS elevation. While the CP analysis allows for a direct elevation comparison against standard coastal survey methods, it provides little information on spatial patterns or differences between the elevation models.

**Table 1:** Survey method elevation difference calculations in comparison to the RTK-GPS. All elevations are in m; NAVD88.

Checkpoints	RTK-GPS elevations (Z)	DEM derived elevations (Z_1_)		Elevation difference (m)	
		sUAS-SfM	Apple lidar	sUAS-SfM	Apple lidar
**July 2023**					
CP 1	178.114	178.098	178.085	−0.015	−0.11
CP 2	177.438	177.397	177.357	−0.039	−0.044
CP 3	177.228	177.21	177.152	−0.023	−0.085
CP 4	177.513	177.471	177.442	−0.048	−0.058
CP 5	178.058	178.038	178.03	−0.021	−0.03
CP 6	177.35	177.338	177.342	−0.046	0.002
CP 7	177.278	177.274	177.207	0.089	−0.065
CP 8	178.811	178.816	178.894	0.006	−0.05
*Average*				−0.012	−0.055
**September 2023**					
CP 1	177.741	177.759	177.739	0.018	−0.002
CP 2	177.665	177.616	177.579	−0.049	−0.086
CP 3	177.862	177.879	177.851	0.017	−0.011
CP 4	177.713	177.705	177.687	−0.008	−0.026
CP 5	177.55	177.56	177.66	0.01	0.11
CP 6	178.14	178.138	178.104	−0.002	−0.036
CP 7	177.615	177.638	177.601	0.023	−0.014
CP 8	177.39	177.41	177.275	0.02	−0.115
*Average*				0.004	−0.022

**Figure 4:**
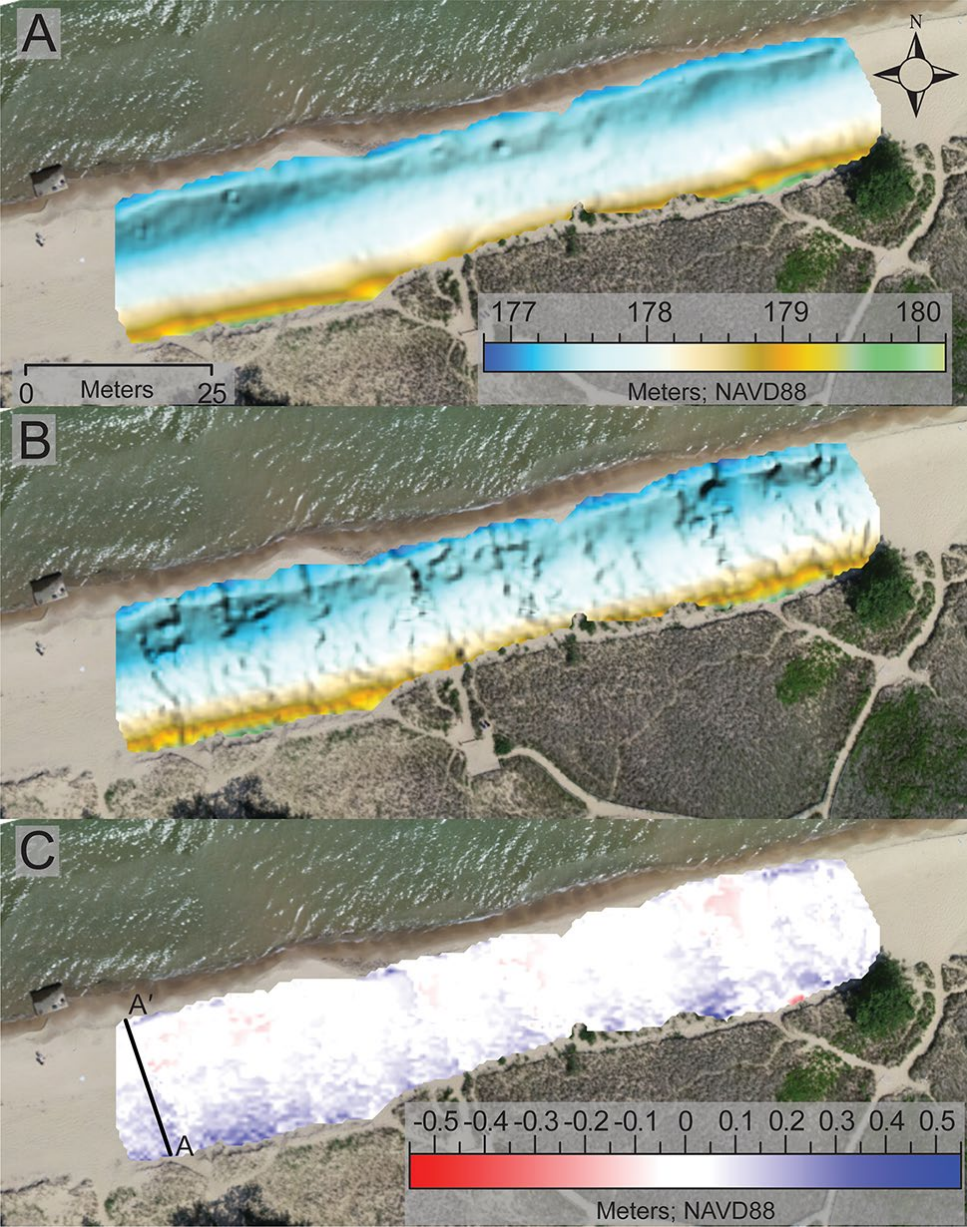
DEMs derived from sUAS-SfM SfM (**A**), the Apple lidar (**B**), and DOD (**C**) derived from the July DEMs represented in this figure. All DEMS are overlaid on an orthomosaic image from 07/18/2023.

**Figure 5:**
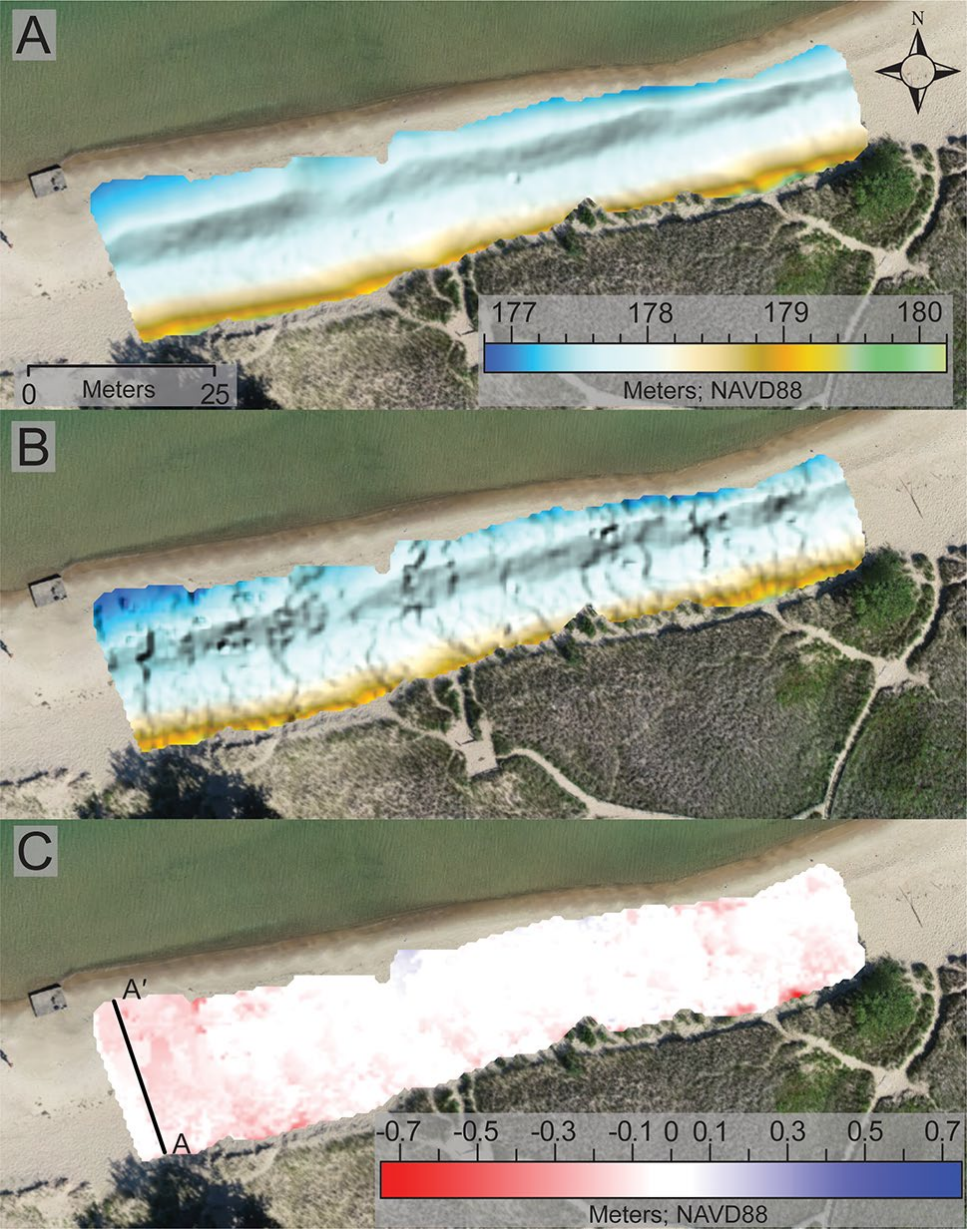
DEMs derived from sUAS-SfM (**A**), the Apple lidar (**B**), and DOD (**C**) derived from the September DEMs represented in this figure. All DEMS are overlaid on an orthomosaic image from 09/01/2023.

To explore the spatial variability in topographic data derived by both sUAS-SfM and Apple lidar we first qualitatively compared the DEMS produced by both methods (Figs. 4 and 5). This was conducted for both the July and September scans. Visually it is clear that the sUAS-SfM method yields a much smoother DEM while the Apple lidar produced a more rugged textured DEM. Some of this rugged character appears to be spatially related to areas where the individual scans were stitched together (i.e., on the edges of the scans). The surface texture detail appears to be the greatest difference between the DEMs derived from the two methods as the general pattern of the elevation contours appear to be nearly identical (Figs. 4 and 5).

Building on this qualitative examination of the DEMs, we compared the spatial statistics calculated within Surfer for both methods and survey dates. The elevation measurements derived from both the Apple lidar and sUAS-SfM were similar for both the July and September datasets. In July, the average elevations for the Apple lidar and sUAS-SfM DEMs were 177.869 m (std. dev. = 0.0683) and 177.79 m (std. dev. = 0.686), respectively. For the September DEMs, the average elevations were 177.854 m (std. dev. = 0.484) for the Apple LIDAR and 177.902 m (std. dev. = 0.492) for the sUAS-SfM.

To further examine the spatial differences between these methods we subtracted the two DEMs for each survey date to evaluate the spatial patterns in elevation offset. Areas that are white in these DODs indicate nearly identical (within 5 cm of each other) elevation measurements. Regions of the survey area that are blue and red show the differences in elevation by the survey method. These areas predominantly occur where the system is more dynamic, such as near the shoreline, foredune, and high foot traffic areas. In areas mapped as blue, the Apple lidar measures a higher elevation compared to the elevation measured by sUAS-SfM. Conversely, in red-mapped areas, the Apple lidar registers a lower elevation than the measurements by sUAS-SfMs. In addition to visually appearing similar, the statistics from the DODs quantitatively reveal that the derived elevations are alike between the two methods. For July, the average elevation difference between the two methods was 0.045 m (std. dev. = 0.076), which is below the vertical change detection threshold, and for September, the difference is −0.058 m (std. dev = 0.068).

**Figure 6:**
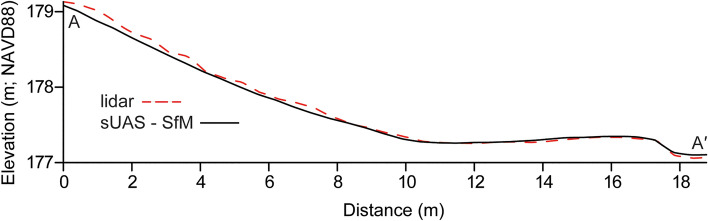
Cross section profile of July lidar and sUAS-SfM DEMs. From 0–10 m (cross-shore distance), the lidar averaged 0.058 m above the sUAS-SfM. Between 10–18 m, the average difference was 0.012 m.

**Figure 7:**
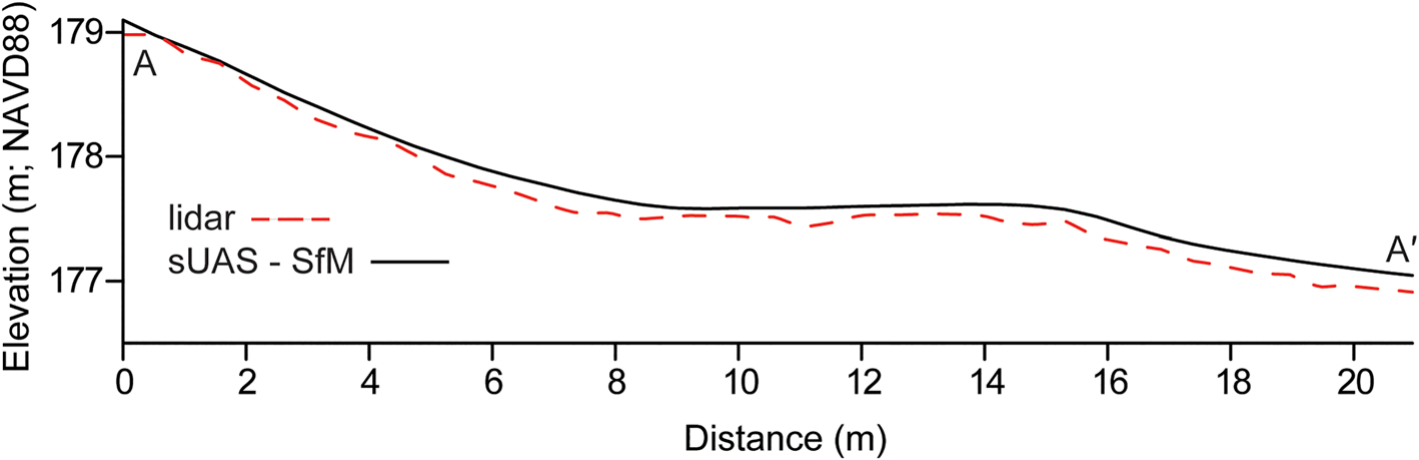
Cross section profile of September lidar and sUAS-SfM DEMs. Apple lidar measured below sUAS-SfM the profile by about -0.099 m on average.

This congruence between methods is further represented in the cross-sectional profiles that were derived from the same position across both the Apple lidar-derived and sUAS-SfM-derived topographic datasets (Figs. 6 and 7). These were generated for both the July and September surveys and allow for a direct comparison of the elevation and profile morphology measurements from both methods. They highlight the similarity of elevation measurements between the two survey methods and illustrate that both methods can accurately capture the beach and foredune morphology. The Apple lidar consistently records a higher elevation than the sUAS-SfM-derived elevation on the foredune (0–10 m cross shore distance) in July, with an average difference of 0.058 m but is nearly identical across the backshore and foreshore, differing by an average of −0.012 (10–18 + m) for an overall average of 0.025 m. For the September survey, the Apple lidar generated elevation data that were consistently lower than the sUAS-SfM by roughly −0.099 m. The elevation measured along these profiles varied the most by 0.138 m in July and −0.167 m in September, with the minimum differences being below the 0.05 m vertical change detection threshold.

After establishing the accuracy of the Apple lidar method in comparison to RTK-GPS and sUAS-SfM-derived elevation data, the ability for the Apple lidar to detect beach and dune geomorphic change over time was explored. DODs generated by differencing the July and September Apple lidar DEMs revealed foreshore accretion throughout the site in response to berm formation (Fig. 7) and additional patchy areas of erosion were observed along portions of the foredune. Foreshore accretion and berm formation was also documented in the sUAS-SfM-derived DOD map, but the patches of foredune accretion were not captured by this method. Only a small patch of foreshore erosion was observed in the sUAS-SfM DOD, but several patches were observed in the Apple lidar DOD.

Both survey methods detected net deposition, due to berm formation, across the study site with the sUAS-SfM averaging 0.116 m (std. dev. = 0.133, RMS = 0.176) of sediment gain and the Apple lidar averaging 0.07 m (std. dev. = 0.133, RMS = 0.176). These average elevation values were generated by Surfer and represent the average of all the grid nodes for the DODs.

The geomorphic changes between July and September were further evaluated by examining volumetric data generated by both methods. The (planar area) normalized volume data were used as the comparison metric between the two methods. Overall, both methods produced similar volumetric measurements with the positive, negative, and net change values being 0.173 m^3^/m^2^, 0.096 m^3^/m^2^, and 0.073 m^3^/m^2^ for the Apple lidar and 0.147 m^3^/m^2^, 0.039 m^3^/m^2^, and 0.120 m^3^/m^2^ for the sUAS-SfM (Table 2). Similar to what was observed in the DOD maps, the Apple lidar measured higher volumetric values of accretion (positive change) and erosion (negative change). These volumetric measurements allow for a quantitative comparison of the spatial patterns that were observed in the DOD maps.

**Table 2:** Volume and area calculations based off sUAS-SfM and Apple lidar Fig. 8 DODs. The normalized volume data (m^3^/m^2^) denotes the amount of sediment gained or lost in a given area. A positive volume indicates sediment deposition and a negative volume indicates erosion.

Survey method	Positive volume (m^3^)	Negative volume (m^3^)	Net volume (m^3^)
*sUAS-SfM*	214.435	9.607	204.829
*Apple lidar*	183.977	60.013	123.964

	Positive planar area (m^2^)	Negative planar area (m^2^)	Net area (m^2^)
*sUAS-SfM*	1454.837	245.413	1700.250
*Apple lidar*	1066.048	627.648	1693.695

	Positive (m^3^/m^2^)	Negative (m^3^/m^2^)	Net (m^3^/m^2^)
*sUAS-SfM*	0.147	0.039	0.120
*Apple idar*	0.173	0.096	0.073

**Figure 8:**
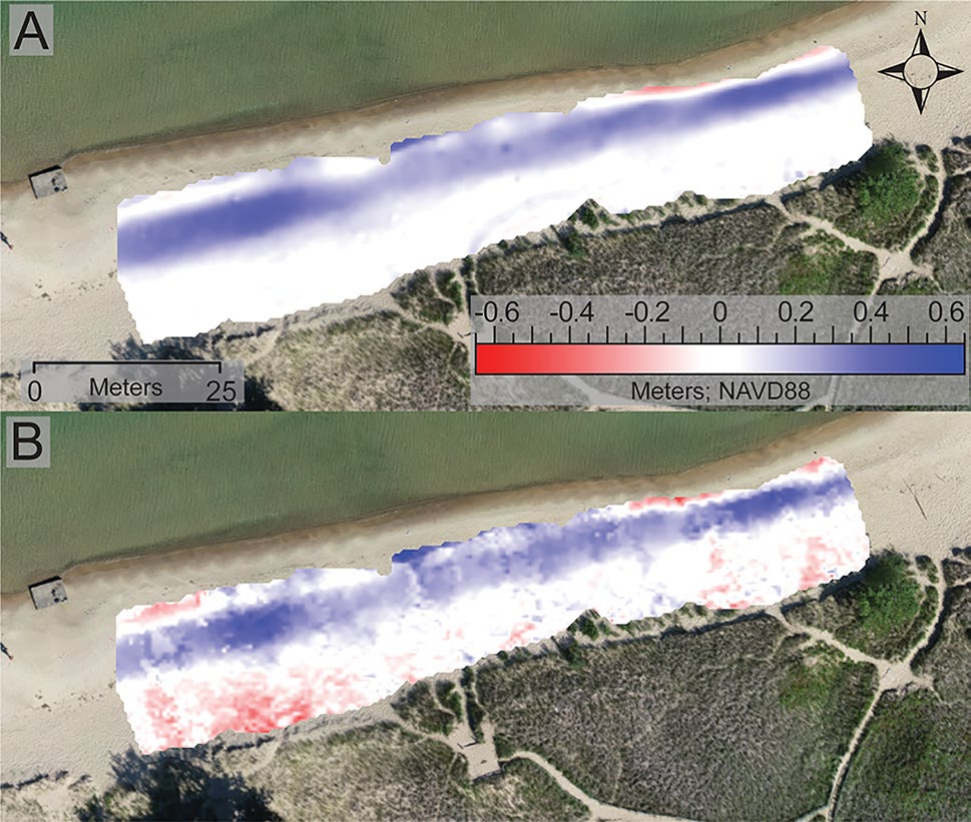
(**A**) DOD map generated from the sUAS-SfM-derived DEMS from 07/18/2023 and 09/01/2023. (**B**) DOD map generated from the Apple lidar-derived DEMs. Both DODs are draped over orthomosaic imagery captured by the sUAS-SfM on 09/01/2023. DODs illustrate areas of erosion, depicted in red, and deposition, shown in blue. Areas with no elevation change are white. Both DODs have a minimum contour of -0.65 m and a maximum contour of 0.65 m, with a contour interval of 0.2 m.

There are differences in erosive and depositional areas between the two DODs, but the large geomorphic change attributes, like the berm, are similar across both models. Within the Apple lidar DOD, two pockets of erosion were detected near the shoreline, but only one of these were captured in the sUAS-SfM DOD. Patchy erosion is also evident in the Apple lidar DOD within the foredune area, but not present at all in the sUAS-SfM DOD. This is most prominent along the SW corner of the foredune, but there are also a couple patches of erosion along the NE stretch of the foredune. Overall, both methods captured the broad spatial patterns of erosion and accretion but the Apple lidar detected more topographic change than the sUAS-SfM.

## Discussion

The Apple lidar has been used in a variety of studies within the environmental sciences^10,30–33^, but the accuracy and capability of the Apple lidar for mapping sandy beach and foredune environments had not been examined. In our study, we first quantitatively compared the accuracy of the Apple lidar and sUAS-SfM to the RTK-GPS surveyed elevations. Once the accuracy of the Apple lidar was established we then compared volumetric measurements and the spatiotemporal patterns of erosion and accretion between the two methods. Results suggest that the Apple lidar can be used as an accurate alternative for coastal monitoring; particularly in areas where sUAS-SfM surveying might be challenging or not allowed.

The spatial comparisons between the sUAS-SfM and Apple lidar survey methods reveal both similarities and differences across the various models. The color ramp for the DEMs, and the large geomorphic features in the DODs suggest that the two survey methods are measuring the same geomorphology. However, the ruggedness of the Apple lidar’s DEMs and some of the smaller scale spatial variations in erosion and accretion documented in the Apple lidar DOD do not align with models derived from sUAS-SfM. The more rugged character of the Apple lidar DEMs may be attributed to several factors that will be discussed below.

First, if the sUAS was flown at a height lower than what was flown in this study (~ 55 m), a lower cm/pixel resolution would be achieved and more detail of the sandy contours could be captured, potentially resulting in the sUAS-SfM model appearing more rugged. Additionally, the Apple lidar point clouds are denser than those from sUAS-SfM, with the scanner generating in total 83,179,489 points in July and 63,984,235 points in September, compared to the sUAS-SfM point clouds which had 6,976,757 points in July and 7,179,555 points in September (Supplemental Table S2). The higher density of the Apple lidar point clouds enhances the morphologic detail that is captured with the survey^36^. This is particularly true in areas such as the foredunes where short grasses occur on top of the bare sand, which is a documented challenge for rendering accurate bare earth DEMs using sUAS-SfM surveying methods^37^. It is possible that the Apple lidar may be able to render a more accurate bare earth DEM in these minimally vegetated areas than sUAS, however, additional processing of the point cloud would be needed to ensure removal of vegetation. The Apple lidar may be a useful alternative to terrestrial laser scanning and airborne lidar for researchers that are focused on ecogeomorphic changes in foredune environments, particularly related to aeolian transport and other fine-scale geomorphic processes^38–40^. Additional work is needed to document the capabilities and limitations of using the Apple lidar to map bare earth in these vegetated areas.

While our study clearly demonstrates utility of the Apple lidar as an alternative method to sUAS-SfM for generating full-coverage beach and foredune DEMs, it also reveals potential for gathering other less spatially comprehensive datasets that might be useful for long-term monitoring or rapid change assessments. The Apple lidar-derived cross-sectional profile generated similar elevation and morphology data to the sUAS-SfM-derived profile (July = 0.025 m, September = −0.099 m) and thus it can be inferred that the Apple lidar could be employed to derive profile data only. Beach profiling is often a more practical method for long-term coastal monitoring yet are limited in the alongshore spatial detail they provide^41,42^. Using the Apple lidar as an alternate method for deriving profiles strikes a balance between efficiently generating the cross-shore topographic information, but also allows for some accounting of the alongshore variability at the station given the way the data are generated (iPad scans side to side as the surveyor walks across the beach). This is a potential surveying option for large stretches of the coast where profiling would generally be the appropriate solution, but some additional 3D data could be beneficial. Furthermore, the ease at which this method can be employed lends itself to being used in community science coastal change monitoring programs, which are gaining popularity throughout the world, particularly using cutting-edge technology such as sUAS-SfM^43,44^.

Beach and foredune morphodynamics dictate the physical vulnerability of coastal areas to hazards, such as sea level rise and storms. As people continue to develop and populate low elevation coastal zones, which include sandy beaches and foredunes^45^, hazard risk and exposure increases^46^. These coastal hazards could lead to costly environmental and socio-economic challenges, with an estimated US$1 trillion in potential economic impacts^47,48^. To proactively address these hazards, coastal decisionmakers need robust scientific information that is guided by high quality data, such as the geomorphic change measurements gathered in this study. However, in many locations sUAS-SfM is not a data collection option due to license, permit, cost, and sometimes weather. However, since it is a handheld device the Apple lidar can be used without these constraints and can be used on populated beaches or in no-fly zones to accurately generate topographicy data.

Sandy beaches and foredunes are incredibly dynamic systems, therefore requiring mapping tools that allow for efficient and accurate mapping of these coastal areas. The results from our study indicate that the Apple lidar is a powerful tool in this context, providing scientists and practitioners with greater accessibility to collect high resolution beach and dune mapping products. Rapid generation of these data products is important for making management decisions as changes in coastal environments can occur suddenly in response to storms and other hazards and managers need information quickly to make informed decisions. Our work, in combination with other studies (e.g.,^10^) suggests that data and information gathered with the Apple lidar could be incorporated into resilience planning to proactively protect coastal assets and adapt to changing conditions.

## Conclusion

The methods developed in this study show promise for future mapping applications that could be ecologically and economically important for sandy beach and foredune landscapes throughout the world. The Apple lidar, a novel tool in coastal science, balances the cost, resolution, and logistical constraints of data collection in comparison to sUAS-SfM making it an effective and accessible approach for monitoring coastal geomorphic change over time. Georeferenced lidar point clouds contain substantially more data than point clouds from sUAS-SfM, which appears to enhance the detail of the elevation models by capturing smaller-scale erosional and depositional features. The accessibility of this new method allows for rapid deployment before and after high-energy storm events as well as other events such as management actions (e.g. nourishment) or water level fluctuations (e.g., high-tide flooding, seiches). The ability to quickly map geomorphic changes resulting from coastal processes is becoming increasingly important and decisionmakers can utilize the Apple lidar to help build resilient management strategies and preserve coastal communities.

### Supplementary Information

Supplementary Information.

## Data Availability

All data from this study are maintained on Michigan State University servers and can be supplied to interested individuals by written request to the corresponding author.
