# Supplementary material for: Comparative analysis of sandy beach and foredune geomorphic change measurements from Apple lidar and small-unoccupied aerial systems
Source: Sci Rep. 2024 Jun 4;14:12828. doi: 10.1038/s41598-024-63466-1 (PMC11150472; doi:10.1038/s41598-024-63466-1)
Supplement: Supplementary file 1 — Supplementary Information. [file 41598_2024_63466_MOESM1_ESM.docx]

*Scientific Reports*

Supplemental Figures and Tables for

**Comparative analysis of sandy beach and foredune geomorphic change measurements from Apple Lidar and small-unoccupied aerial systems**

**Brendan M.J. Burchi^1^ and Ethan J. Theuerkauf^1*^**

^1^ Department of Geography, Environment, and Spatial Sciences, Michigan State University, 673 Auditorium Road, East Lansing, MI 48824.

Corresponding author: Ethan J. Theuerkauf ([theuerk5@msu.edu)](mailto:theuerk5@msu.edu))


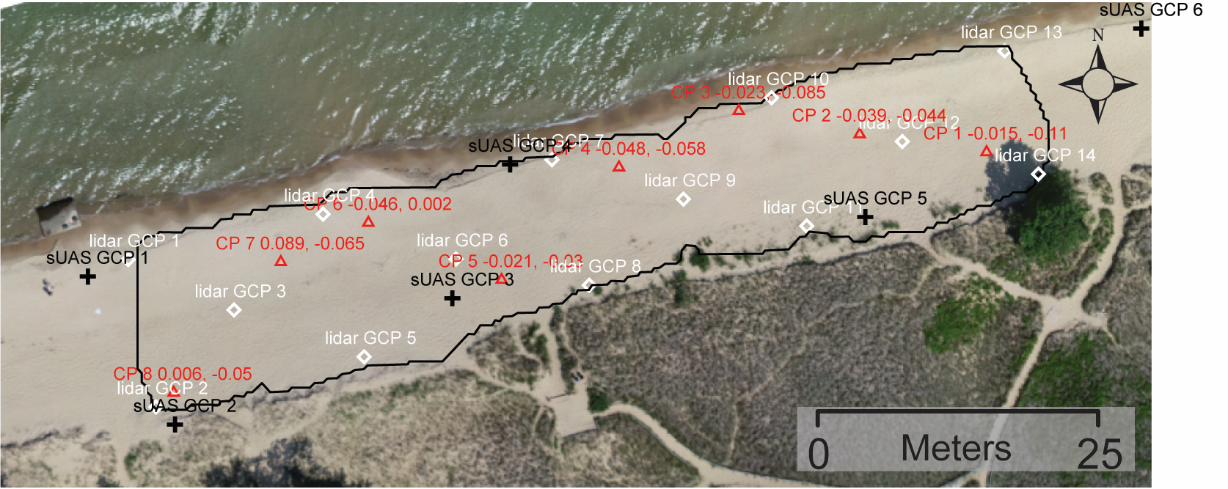


Supplementary Figure S1. Location of Apple lidar and sUAS GCPs for field data collection in July. The lidar GCPs are white diamonds and the black crosses are sUAS GCPs. The black polyline is the final bounding box for the DEM used for the July analysis. CPs are red triangles and have the difference in elevations of the survey method to the RTK-GPS (sUAS, Apple lidar).


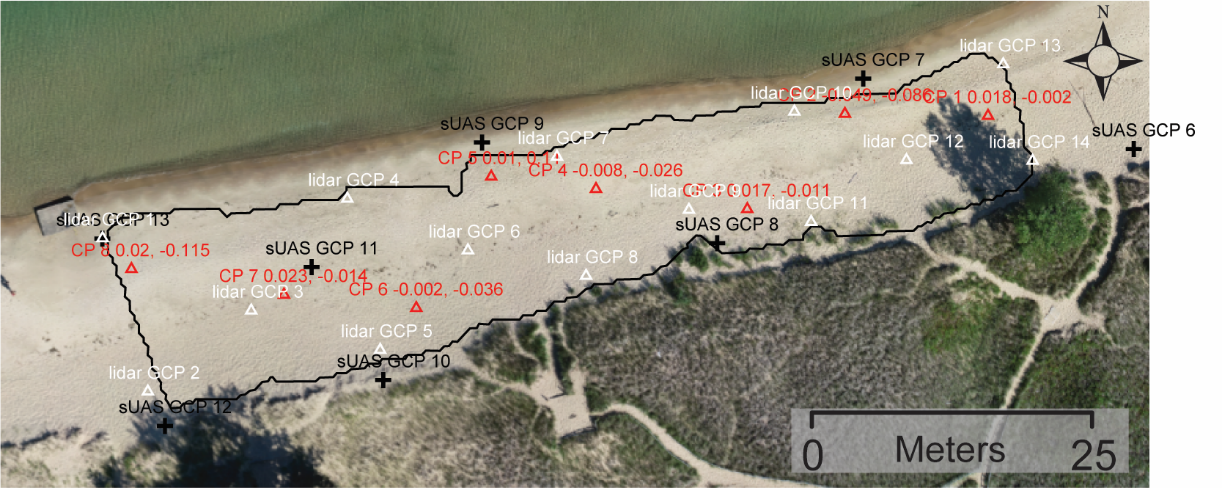


Supplementary Figure S2. Location of Apple lidar and sUAS GCPs for field data collection in September. The lidar GCPs are white diamonds and the black crosses are sUAS GCPs. The black polyline is the final bounding box for the DEM used for the July analysis. CPs are red triangles and have the difference in elevations of the survey method to the RTK-GPS (sUAS, Apple lidar).

| Measurer | Dimensions | Observed (cm) | Actual (cm) | Similarity |
| --- | --- | --- | --- | --- |
| 1 | Length | 42.3 | 45.5 | 92.97% |
|  | Width | 38.9 | 41 | 94.88% |
|  | Height | 29.78 | 31.5 | 94.54% |
| 2 | Length | 42.9 | 45.5 | 94.29% |
|  | Width | 39 | 41 | 95.12% |
|  | Height | 29.99 | 31.5 | 95.21% |
| 3 | Length | 41.7 | 45.5 | 91.65% |
|  | Width | 37.3 | 41 | 90.98% |
|  | Height | 28.89 | 31.5 | 91.71% |
| 4 | Length | 44.2 | 45.5 | 97.14% |
|  | Width | 38.9 | 41 | 94.88% |
|  | Height | 29.72 | 31.5 | 94.35% |
|  |  |  |  | Average |
|  |  |  |  | 93.98% |

Supplementary Table S1. Observed and actual length, width, and height measurements of a rectangular box measured within the 3D scanner app (observed) and tape measure (actual). The similarity of the observed and actual values averages above a 90%.

|  |  |  |  |
| --- | --- | --- | --- |
|  |  | Apple Lidar |  |
| Scans | July Data Points |  | September Data Points |
| 1 | 9,338,757 |  | 11,403,513 |
| 2 | 11,760,441 |  | 11,116,705 |
| 3 | 12,157,210 |  | 8,737,422 |
| 4 | 12,711,152 |  | 7,879,578 |
| 5 | 10,450,347 |  | 7,728,449 |
| 6 | 8,573,712 |  | 8,127,678 |
| 7 | 12,558,170 |  | 8,343,066 |
| 8 | 12,609,457 |  | 7,827,379 |
| Total: | 90,159,246 |  | 71,163,790 |
|  |  |  |  |
|  |  | sUAS-SfM |  |
|  | July Data Points |  | September Data Points |
| Total: | 6,976,757 |  | 7,179,555 |

Supplementary Table S2. Total processed point cloud data for Apple lidar and sUAS-SfM. Each Apple lidar scan contained more point cloud data than the total sUAS-SfM point cloud for both survey dates.
